# Gold nanoparticle-mediated laser stimulation induces a complex stress response in neuronal cells

**DOI:** 10.1038/s41598-018-24908-9

**Published:** 2018-04-25

**Authors:** Sonja Johannsmeier, Patrick Heeger, Mitsuhiro Terakawa, Stefan Kalies, Alexander Heisterkamp, Tammo Ripken, Dag Heinemann

**Affiliations:** 1 Industrial and Biomedical Optics Department, Laser Zentrum Hannover e.V, Hollerithallee 8, 30419 Hannover, Germany; 2 Cluster of Excellence “Hearing4All”, Hannover, Germany; 3 Institute of quantum optics, Gottfried Wilhelm Leibniz Universität Hannover, Welfengarten 1, 30167 Hannover, Germany; 4 Lower Saxony Centre for Biomedical Engineering, Implant Research and Development (NIFE), Stadtfelddamm 34, 30625 Hannover, Germany; 5 School of Integrated Design Engineering, Keio University, 3–14-1 Hiyoshi, Kohoku-ku, Yokohama 223-8522 Japan; 6 Department of Electronics and Electrical Engineering, Keio University, 3–14-1 Hiyoshi, Kohoku-ku, Yokohama 223-8522 Japan

## Abstract

Stimulation of neuronal cells generally resorts to electric signals. Recent advances in laser-based stimulation methods could present an alternative with superior spatiotemporal resolution. The avoidance of electronic crosstalk makes these methods attractive for *in vivo* therapeutic application. In particular, nano-mediators, such as gold nanoparticles, can be used to transfer the energy from a laser pulse to the cell membrane and subsequently activate excitable cells. Although the underlying mechanisms of neuronal activation have been widely unraveled, the overall effect on the targeted cell is not understood. Little is known about the physiological and pathophysiological impact of a laser pulse targeted onto nanoabsorbers on the cell membrane. Here, we analyzed the reaction of the neuronal murine cell line Neuro-2A and murine primary cortical neurons to gold nanoparticle mediated laser stimulation. Our study reveals a severe, complex and cell-type independent stress response after laser irradiation, emphasizing the need for a thorough assessment of this approach’s efficacy and safety.

## Introduction

Laser-based neuronal stimulation has opened up a new and promising field of research. Using a laser pulse instead of an electrical signal, the stimulus can be placed with unprecedented spatial resolution since electronic crosstalk is avoided. The light pulse can be converted to an adequate stimulus for a neuron by introducing light-sensing ion channels (optogenetics)^1^, photocleavable chemical compounds^2,3^ or by using exo- or endogenous photoabsorbers. In the case of photoabsorbers, it has been shown that the resulting rapid temperature transient increases the cell’s membrane capacitance, generating depolarizing currents that may trigger action potentials in nerve cells^4,5^. Water in and around the cell is the main absorber for direct infrared stimulation. However, bulk-heating of the irradiated area comprises the precision and selectivity of the stimulus. Nanoabsorbers like gold nanoparticles present a more specific alternative: When irradiated at their plasmon resonance, gold nanoparticles heat up rapidly and confer this heat to the plasma membrane^5^. The particles’ resonance frequency can be tuned by varying their geometrical characteristics, such as aspect ratio, or the shell-core composition. Spherical gold nanoparticles have an absorption maximum of approx. 530 nm, increasing with diameter^6^. Activation of neuronal cells has been demonstrated with both spherical^5,7^ and cylindrical^8^ gold nanostructures. Translating this stimulation mechanism to neural implants such as deep brain stimulators or cochlear implants could potentially reduce adverse effects that arise from imprecise electrical stimuli without the need for genetic modification, which is required in optogenetics. However, it is still widely unknown to what extent gold nanoparticle mediated laser stimulation interferes with the cellular metabolism and physiology. Depending on the parameters used for irradiation, rapid and substantial heating of gold nanoparticles may also cause protein denaturation, shockwaves and evaporation^9^. Next to reliable cell activation, biological safety is crucial for a neural implant. Therefore, a fundamental assessment of the method’s biological impact is essential before laser-based neurostimulation can be safely transferred to use in animal or human trials.

In order to investigate the impact of gold nanoparticle-meditated laser stimulation, we focused on calcium as a general marker of cellular stress signaling. Calcium is a universal messenger that is involved in numerous signaling pathways^10,11^. It plays a particularly important role in mediating stress responses and inducing apoptosis^12–14^. Tracking the calcium flux within a stimulated cell can provide information on various aspects of its reactions and health. The use of fluorescent calcium sensors allows monitoring of events like action potentials or stress responses in parallel to laser stimulation^7,15,16^. To gain information about the source of calcium flow, different possible pathways can be blocked one by one with specific inhibitors. In this study, we used fluorescent dyes to monitor intracellular calcium flux, lipid peroxidation events and membrane perforation in response to gold nanoparticle-mediated laser stimulation of the murine Neuro-2A (N2A) cell line and primary mouse cortical neurons. Various inhibitors were used to investigate the involvement of specific pathways. We integrated the information from different endpoints of a large data set to reconstruct the events immediately following a laser stimulus. Our results reveal an unspecific stress response that can be potentiated by calcium influx through membrane pores and intracellular feedback loops. Lipid peroxides and reactive oxygen species (ROS) might play a role as chemical mediators to forward the signal from the membrane into the cell, triggering a global and potentially lethal stress response.

## Methods

### Cell culture

**Figure 1:**
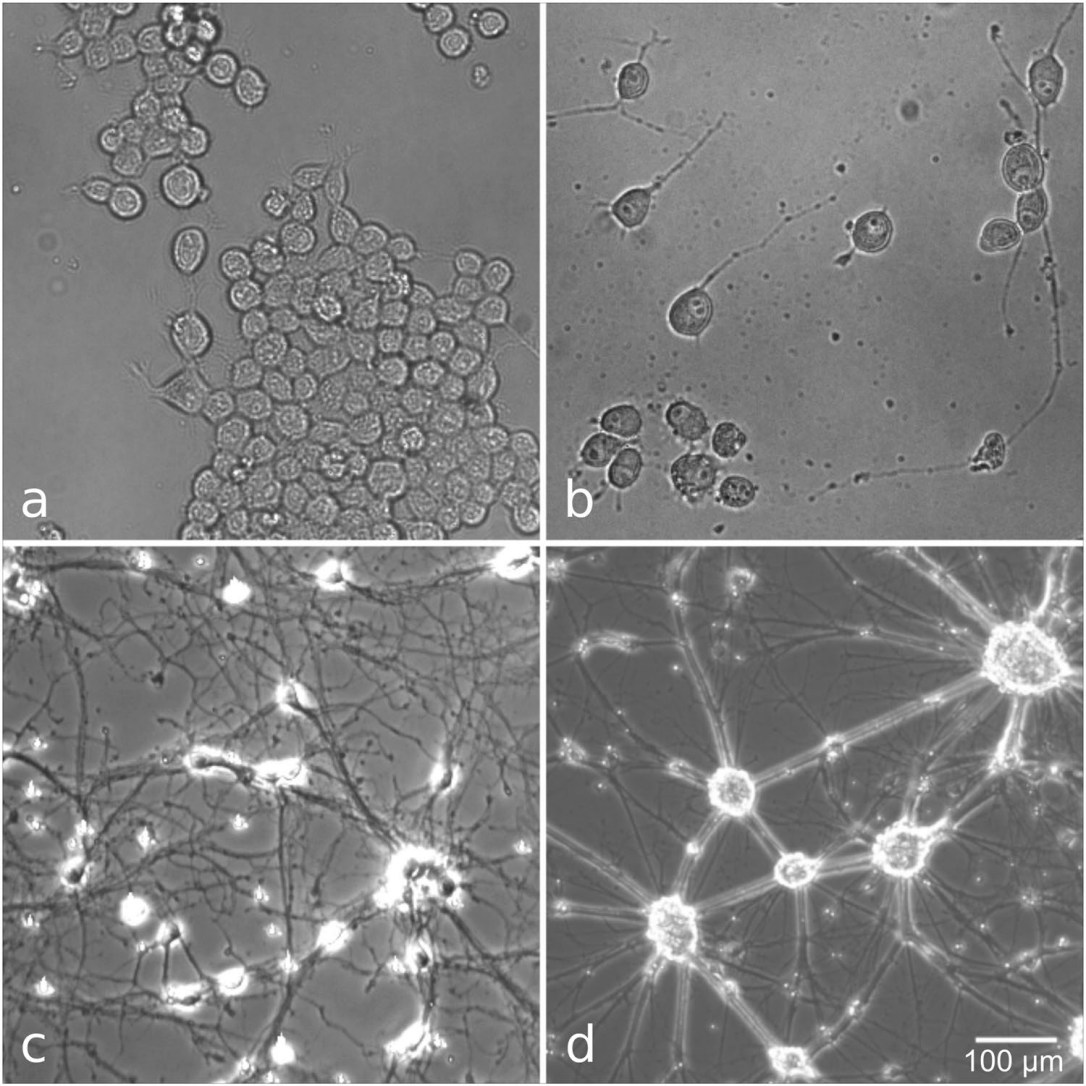
Cell types used in this study. a, b: N2A cells before (**a**) and three days after serum deprivation (**b**). (**c**,**d**) Primary mouse cortical neurons, 7 (**c**) and 14 (**d**) days after start of culture. Mature cells formed dense clusters that did not allow for single cell stimulation. Therefore, neurons were used at the earlier time point. Scale bar corresponds to all panels.

N2A cells were cultured in MEM Eagle with EBSS, supplemented with 10% FCS, 1% Penicillin-Streptomycin and 1% non-essential amino acids (all Pan Biotech, Germany), and incubated at 37 °C, 5% CO_2_. Prior to the experiments, 70,000 cells were seeded in a glass bottom dish (diameter 35 mm, ibidi, Germany). Cells were allowed to attach to the dish and subsequently cultured in serum-free medium for two to three days to promote neurite outgrowth and neuronal differentiation^17^ (Fig. 1a,b).

Mouse cortical neurons (MCN) were obtained from Thermo Fisher Scientific (USA) and cultured according to the supplier’s protocol in 35 mm glass bottom dishes. Neurobasal® Medium supplemented with 0.5 mM Glutamax^TM^-I and 2% B-27® was used as culture medium and refreshed every third day. After one week in culture, the neurons had developed multiple long neurites. Experiments were performed after 7–10 days in culture (Fig. 1c,d).

### Laser manipulation setup

The setup used for single cell manipulation experiments has been described before^15^. A weakly focused pulsed Nd:YAG laser beam (λ = 532 nm, pulse width = 850 ps, repetition rate = 20.25 kHz; Horus, France) was guided onto the sample from above. The spot diameter was approx. 60 µm. An inverse epifluorescence microscope (Axio Observer, HBO 50 fluorescence excitation lamp, HAL100 brightfield lamp) was used to monitor fluorescence signals and obtain bright field images. Images were acquired with a cooled CCD-camera (ProgRes MF cool, Jenoptik, Germany). An OD6 notch filter (NF533-17, Thorlabs) protected the camera from laser irradiation. We used a 480 nm ± 15 nm bandpass filter for Fluo 4 excitation; emission was evaluated with a 520 nm longpass filter.

### Laser stimulation and calcium response

On the day of experiment, cells were incubated for three hours with 200 nm gold nanoparticles (AuNP, Kisker Biotech) at a concentration of 0.5 µg/cm^2^. The same particles have been used before for laser transfection^18,19^, perforation studies and assessment of cellular stress^15,20^. Previous work has shown that after three hours of incubation time, particles have sedimented and are homogeneously distributed across the surface, where they attach to the cells’ membrane. The concentration used here results in 5–7 particles per cell^18^. Cellular uptake plays a negligible role for the size of particles and the time scale used in this study^21^. The medium was then replaced with serum free culture medium containing 1:1 Fluo 4-AM and Pluronic-F127 (5 or 1 µM for N2A cells and MCN, respectively; Sigma-Aldrich, USA). N2A cells and MCN were incubated with the dye for 60 or 30 minutes, respectively. The culture medium was then replaced with the respective experimental medium (either normal culture medium, a buffer, or medium containing an inhibitor; see below) containing propidium iodide (PI, 2 µg/mL, Thermo Fisher Scientific). The dish was placed in the manipulation setup and a 40 ms laser pulse was applied to the targeted cell. Only cells that were not part of a dense cluster (i.e. individual cells) were selected at random for stimulation. Additional cells located in the periphery of a laser spot were not evaluated. Radiant exposures varied from 17 to 51 mJ/cm^2^. Control experiments were conducted for each radiant exposure without the use of AuNP. The evoked calcium response was visualized via Fluo 4 imaging and recorded at five fps with an exposure time of 200 ms at linear gain settings. The sample size per dish was n ≈ 10 cells, and unless noted otherwise, three dishes were tested for each condition (four dishes for N2A cells in culture medium at 25 mJ/cm^2^). Observation time was 30 or 90 s in total, depending on the experimental condition.

### Inhibitors

Stimulation experiments with N2A cells were carried out under the following conditions: culture medium only, phosphate buffered saline (PBS) without Ca^2+^ and Mg^2+^, PBS with Ca^2+^ and Mg^2+^ (PBSC; all PAN-Biotech) or the inhibitors 2-Aminoethoxydiphenyl borate (2-APB), CGP37157 (CGP), lidocaine (lid), ruthenium red (RR; all Cayman Chemical Company, USA), Cyclosporine A (CspA) or ryanodine (Ry; both Enzo Lifesciences, USA) in culture medium. Ryanodine and 2-APB were also used in combination. All inhibitors were stored in a stock solution in DMSO at −20 °C, except for RR, which was prepared in culture medium on the day of experiment.

**Figure 2:**
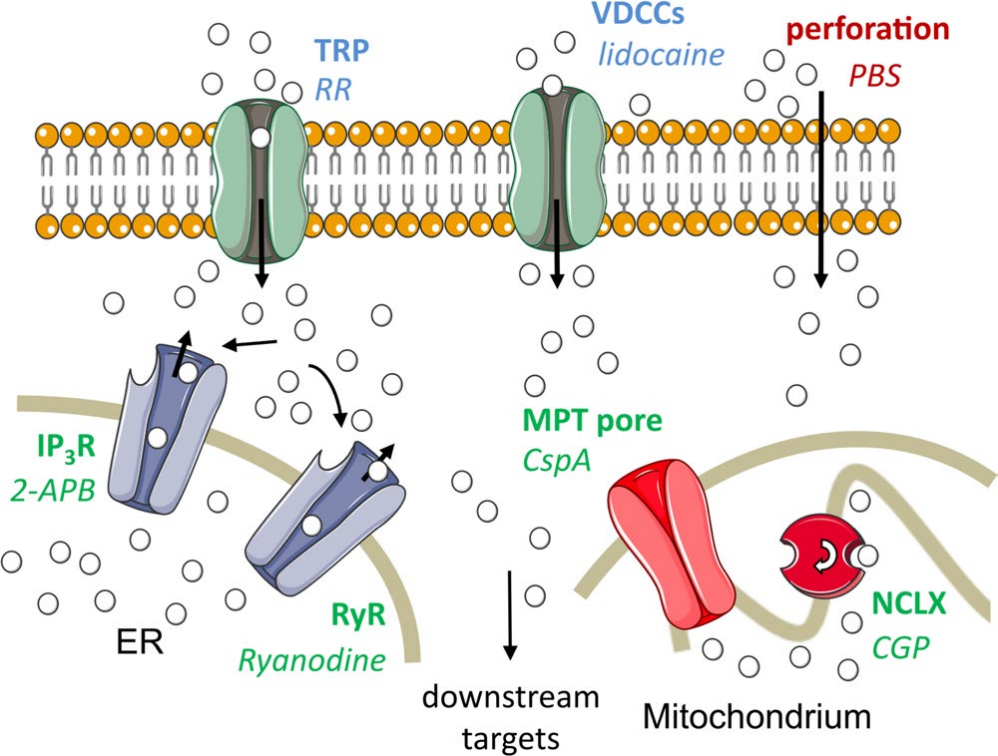
Overview of calcium pathways assessed in this study. Mechanisms of Ca^2+^ transport are given in bold, the respective interfering substance in italics. Intracellular pathways are denoted in green, transport across the cell membrane in blue and non-physiological mechanisms (i.e. cell perforation) in red. In the case of lidocaine, the substance does not interfere directly with the VDCCs but with voltage-dependent sodium channels, which would in turn activate the VDCCs. Calcium ions (white spheres) can be transported to the cytoplasm from the outside medium or intracellular sources, using both physiological and pathophysiological pathways. The MPT pore is closed under physiological conditions. Membrane channels of the ER can be triggered via a positive feedback loop which induces calcium-induced calcium release. TRP, transient receptor potential channels; VDCCs, voltage dependent calcium channels; IP_3_R, inositol trisphosphate receptors; RyR, ryanodine receptors; NCLX, mitochondrial sodium calcium exchanger; ER, endoplasmic reticulum. Figure elements from Servier Medical Art (http://smart.servier.com).

All experiments including inhibitors were conducted at a radiant exposure of 25 mJ/cm^2^. Table 1 gives an overview of the compounds, their final concentration and mode of action. Figure 2 gives a summary of the calcium pathways and the interfering substances.

**Table 1:** Summary of experimental conditions, compound concentrations and cellular targets.

Compound	Abbr.	Conc.	Cellular target	Comment
culture medium	medium	—	—	Physiological condition
PBS w/o Ca^2+^, Mg^2+^	PBS	—	—	Absence of extracellular Ca^2+^
PBS w/ Ca^2+^, Mg^2+^	PBSC	—	—	Control for PBS
2-Aminoethoxy-diphenyl borate	2-APB	75 µM	IP_3_-receptors in the ER membrane^43^	Also interferes with some TRP channels^44^
Ryanodine	Ry	50 µM	Ryanodine receptors in the ER membrane^45^	Also used in combination with 2-APB (50 µM Ry + 100 µM 2-APB)
CGP37157	CGP	20 µM	Mitochondrial Na^+^/Ca^2+^-exchanger^46^	
Ruthenium Red	RR	100 µM	Heat-activated TRP channels^47^	Also blocks intracellular channels, but does not cross the cell membrane^48^
Lidocaine	Lid	100 µM	Voltage-dependent sodium channels^49^	Sodium currents during an action potential would activate voltage-dependent calcium channels^50^
Cyclosporine A	CspA	5 µM	mitochondrial membrane permeability transition pore^35,36^	

Experiments were designed to inhibit specific pathways of cellular calcium signaling as well as study the overall impact of extracellular calcium.

MCN were tested in medium, PBS (one experiment each at 25 mJ/cm^2^) and PBSC (three experiments at 25 mJ/cm^2^, one experiment at 17, 34, 42, and 51 mJ/cm^2^, respectively).

### Lipid peroxidation

The lipid peroxidation sensor BODIPY 581/591 (Thermo Fisher Scientific) was used to visualize lipid peroxidation in the cell membrane caused by laser stimulation. The compound was stored at −20 °C at a concentration of 5 mM in DMSO. Cells were prepared for laser stimulation as described above, and incubated with 10 µM BODIPY 581/591 instead of Fluo 4-AM for 30 minutes at 37 °C. The dishes were then rinsed three times with PBS and the respective test medium was added. Experiments were conducted with N2A cells in culture medium (25 and 51 mJ/cm^2^), PBS and PBSC (51 mJ/cm^2^), and MCN in PBSC (51 mJ/cm^2^), both with and without AuNP. Maximum radiant exposure was chosen to ensure a visible reaction. In its non-oxidized form, BODIPY 581/591 emits red light at 590 nm. Upon oxidation, the emission peak shifts to 520 nm^22^. Due to strong photobleaching by the laser, lipid peroxidation could not be visualized by tracking the fluorescence decrease of the red emitting form. Instead, the fluorescence increase of the green emitting form was used to assess oxidation events using the same setup as described above.

### Data analysis

Images were analyzed with Fiji (ImageJ v.2.35^23,24^). For each cell, the mean gray value was calculated at each time point. Signals recorded after laser stimulation were normalized to baseline fluorescence values. Peak change in fluorescence (max ∆F/F_0_) and the time from laser pulse to peak fluorescence (time to peak) were recorded for each cell. Outliers in each experiment were detected using the interquartile range method. Values that were not included in the interval

1 $${x}_{0.25}-1.5\ast IQR\le {x}_{i}\le {x}_{0.75}+1.5\ast IQR$$

were excluded from analysis unless noted otherwise. Presence or absence of PI-influx was determined by before/after imaging. Proportions that are given without a margin of error were calculated from the pooled data set.

Data analysis and visualization was performed with R (v.3.3.2^25^).

### Data availability

The datasets generated and analyzed during the current study are available from the corresponding author on reasonable request.

## Results

### General calcium response

The general signature of the calcium response was quantified to assess the overall response of the cells under physiological conditions *in vitro*. The magnitude of the response and its time course give a first indication of the underlying mechanism.

**Figure 3:**
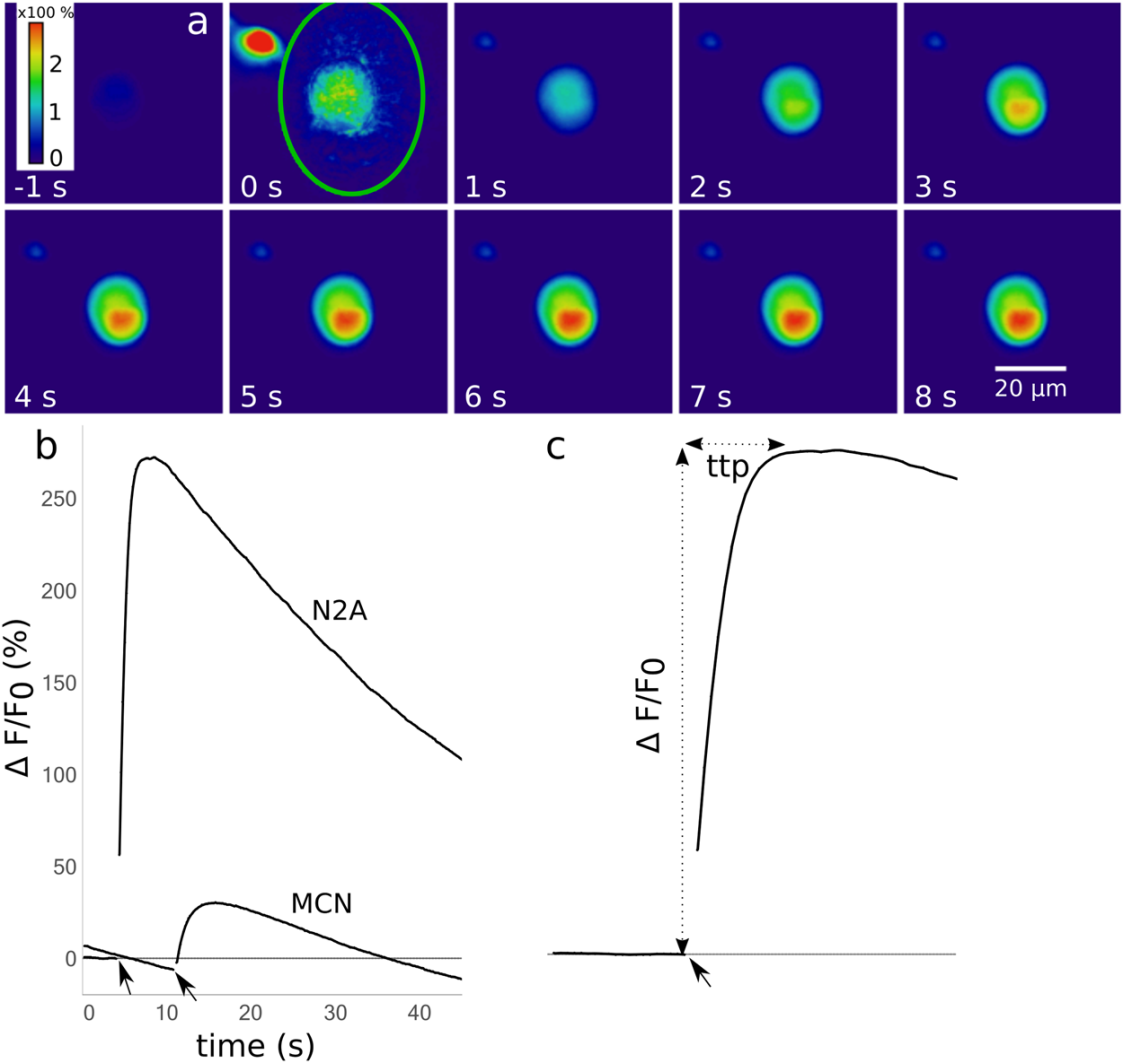
(**a**) Image series of a cell undergoing laser stimulation. The stimulus was placed at 0 s, and images were recorded at 5 fps. The green line at 0 s outlines the laser spot. A second cell (red spot at 0 s) was located in the vicinity of the spot, but not evaluated. The sequence shows the Fluo 4 intensity 1 s before the stimulus and up to 8 s afterwards. Fluorescence increase (%) is color coded. Maximum fluorescence was reached after approx. 6 s. (**b**) Representative depictions of the most frequently observed calcium signatures in N2A cells and MCN, respectively. Arrows indicate where the laser stimulus was placed. The respective frame was excluded from quantification, resulting in a gap in the graphs. (**c**) Illustration of the measurements taken for quantitative evaluation. Maximum increase in fluorescence (ΔF/F_0_) was measured relative to the fluorescence values before laser stimulation. Time to peak (ttp) was defined as the time in s from the laser pulse to the peak value.

**Figure 4:**
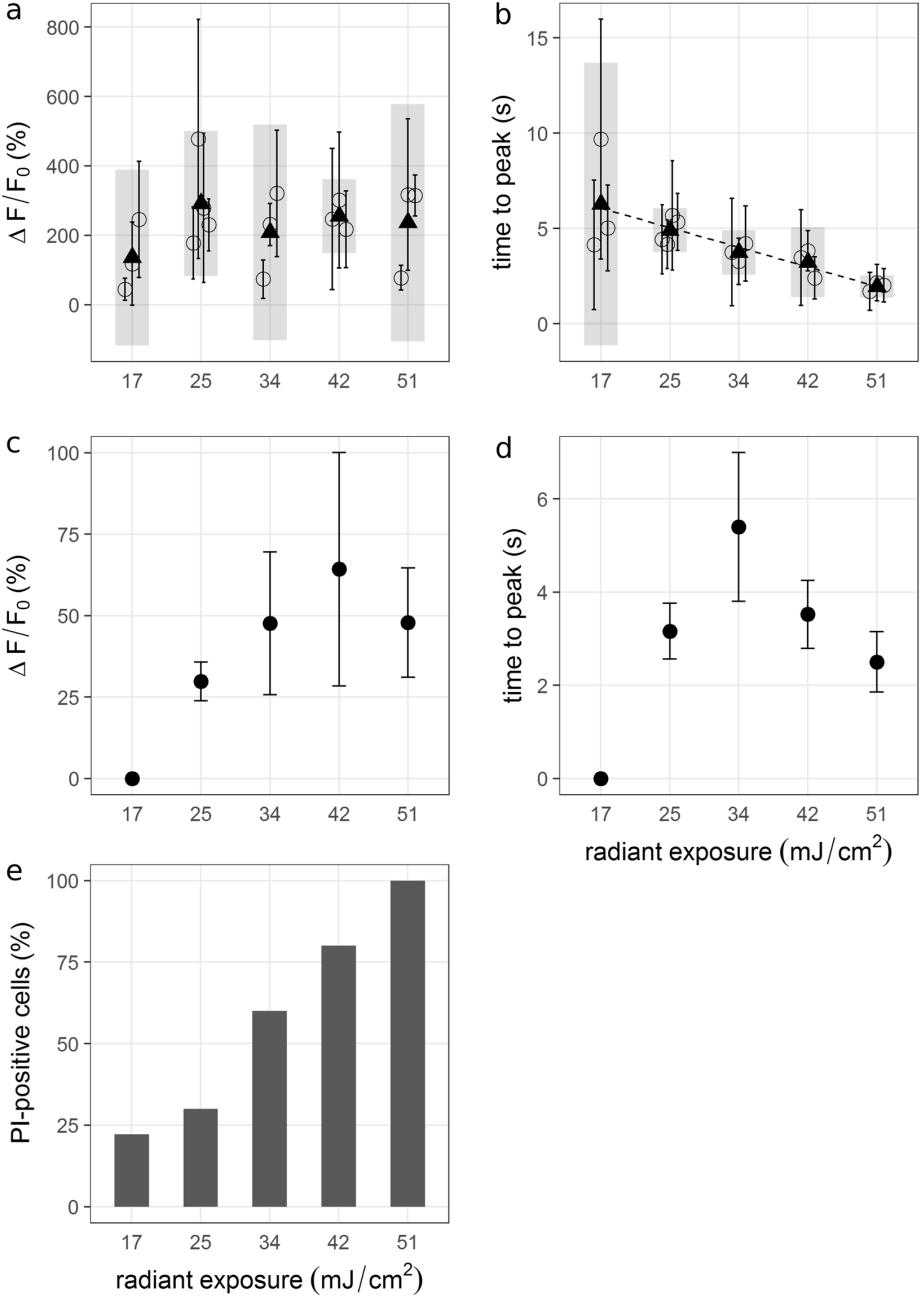
∆F/F0 and ttp values from N2A (**a**,**b**) and MCN (**c**,**d**) versus radiant exposure. (**a**,**b**) Mean values and standard deviations were calculated for each experiment with n = 10 cells (circles + error bars). Black triangles are averages of the respective means per radiant exposure and grey bars are the associated 95%-confidence intervals. While there is no visible trend in ∆F/F_0_ values, time to peak decreased with increasing radiant exposure. (**c**,**d**) Mean values and standard deviations were calculated from one experiment with n = 10 cells. Larger values for radiant exposure tended to yield larger peak fluorescence values, but also resulted in larger variation. Time to peak values do not show a clear trend. MCN were not activated at 17 mJ/cm². (**e**) The proportion of PI-positive N2A cells (pooled) increased with radiant exposure.

**Figure 5:**
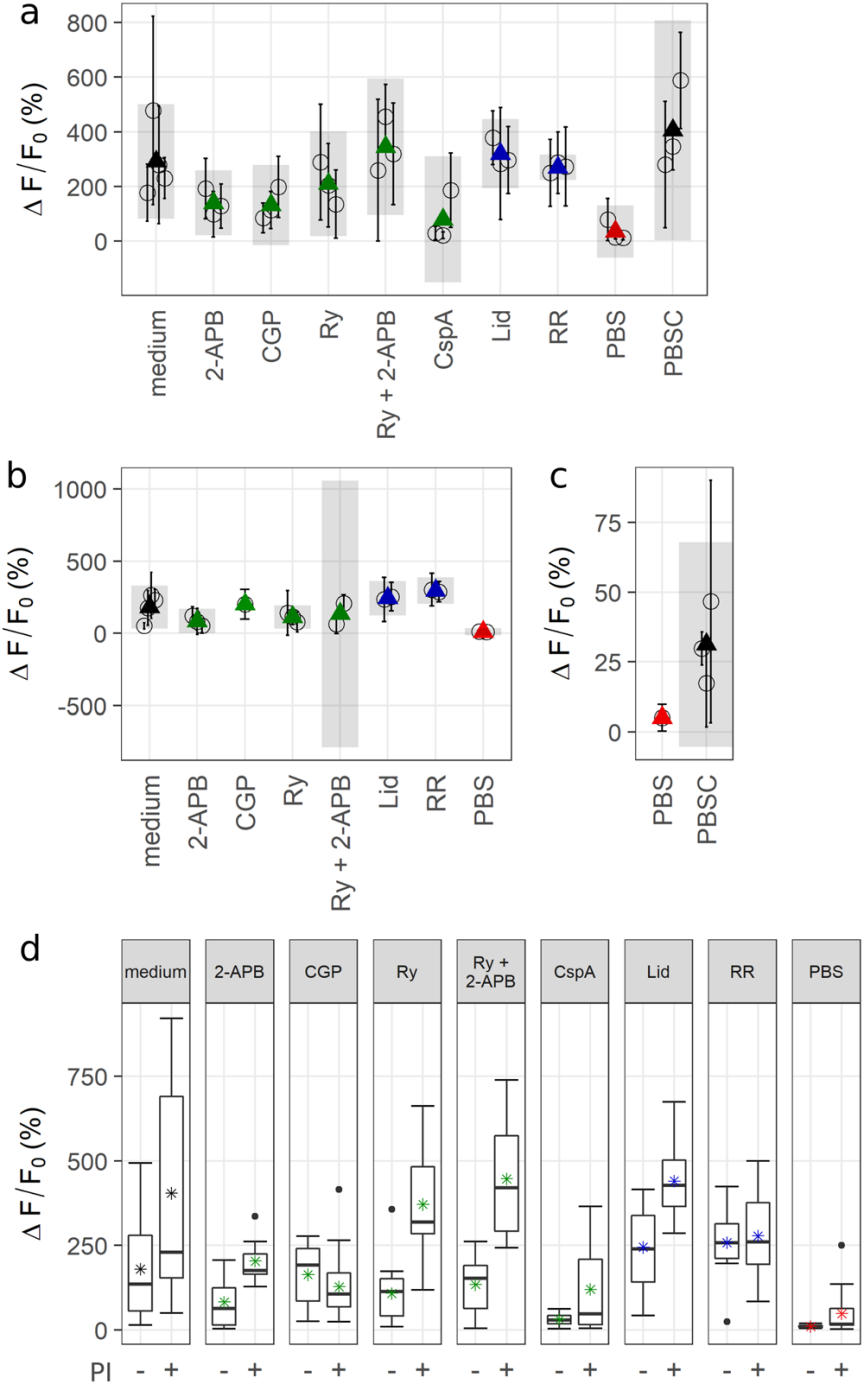
(**a**) Maximum ∆F/F_0_ values of N2A cells plotted by condition. Mean values (circles) and standard deviations were calculated per experiment and averaged for each condition (triangles). Colors refer to the targeted mechanism of calcium transport (see Fig. 2). Grey bars are 95%-confidence intervals of averaged means. Cells in the absence of Ca^2+^ or treated with 2-APB, CGP or CspA exhibited a decreased fluorescence maximum. (**b**) Maximum ∆F/F_0_ values of PI-negative cells. Only experiments with n ≥ 3 responding PI-negative cells were considered. Occurrence of PI-influx gives implications about membrane perforation. A decrease of fluorescence values by CGP is no longer observable. (**c**) Peak fluorescence values of MCN plotted by condition. Absence of extracellular Ca^2+^ markedly decreased the calcium transients. (**d**) PI-negative (−) and positive (+) N2A cells pooled by condition. Some inhibitors caused a distinct determination between PI-positive and negative cells.

Cells that were not pre-treated with AuNP never showed an increase in Fluo 4 fluorescence in response to the laser stimulus. In the presence of AuNP, N2A cells in culture medium (without inhibitors) displayed a fluorescence increase by 100–400% within five to ten seconds, with an average increase of 200%. MCN showed no calcium response in culture medium, but a fluorescence increase by 20–60% (avg. 44%) in PBSC on the same timescale. Qualitatively, the calcium trace was highly reproducible in both cell types. Representative calcium responses are depicted in Fig. 3. The most frequently observed signature (Fig. 3b), which was recorded from 84% of stimulated N2A cells (outliers included), consisted of an immediate rise to a peak value, followed by a slower decay. To assess the impact of photobleaching, images were taken intermittently once every 60 s over 5 minutes. This mode of imaging avoided constant illumination of the fluorescent dye. The fluorescence signal remained stable, suggesting that the decay as depicted in Fig. 3b occurred mainly due to photobleaching. Occasionally (<4% of cells), the laser pulse did not evoke a fast response, but a slower rise that did not peak within observation time (30–90 s). Figure 4 shows the extent of the calcium response and its timescale depending on radiant exposure for N2A cells (a, b) and MCN (c, d). For values >17 mJ/cm^2^, the strength of the calcium response did not depend on the radiant exposure. The proportion of activated cells was consistently high for those values (80–100%). For N2A cells, there was a negative correlation between radiant exposure and time to peak (p < 0.001). Cells from the same experiment were pooled to account for variation between dishes. The respective mean values were averaged for each radiant exposure to estimate the overall mean (see Fig. 4b). The resulting regression line is given by

$$f({H}_{e})=-\,1.03\ast {H}_{e}+7.10,$$

where H_e_ is the radiant exposure. Radiant exposure accounted for 57.4% of variability in mean time to peak values (*R*^2^ = 0.5738). Perforation of the cell membrane during stimulation was assessed by PI-influx. The proportion of PI-positive cells increased with radiant exposure (Fig. 4e). No distinction could be made between PI-positive and negative cells concerning the features of the calcium response, i.e. its shape and values for magnitude of peak and time to peak, except for a larger variation of ∆F/F_0_ of PI-positive cells (see Fig. 5d). The following experiments that included an inhibitor were conducted at 25 mJ/cm^2^. At this value, a high probability of activation was observed together with a relatively low probability of perforation.

### Inhibitor studies

Stimulation experiments were repeated with cells under different conditions. Inhibitors were employed to interfere with cellular calcium pathways (see methods section). All results presented in this section were obtained at a radiant exposure of 25 mJ/cm^2^.

**Figure 6:**
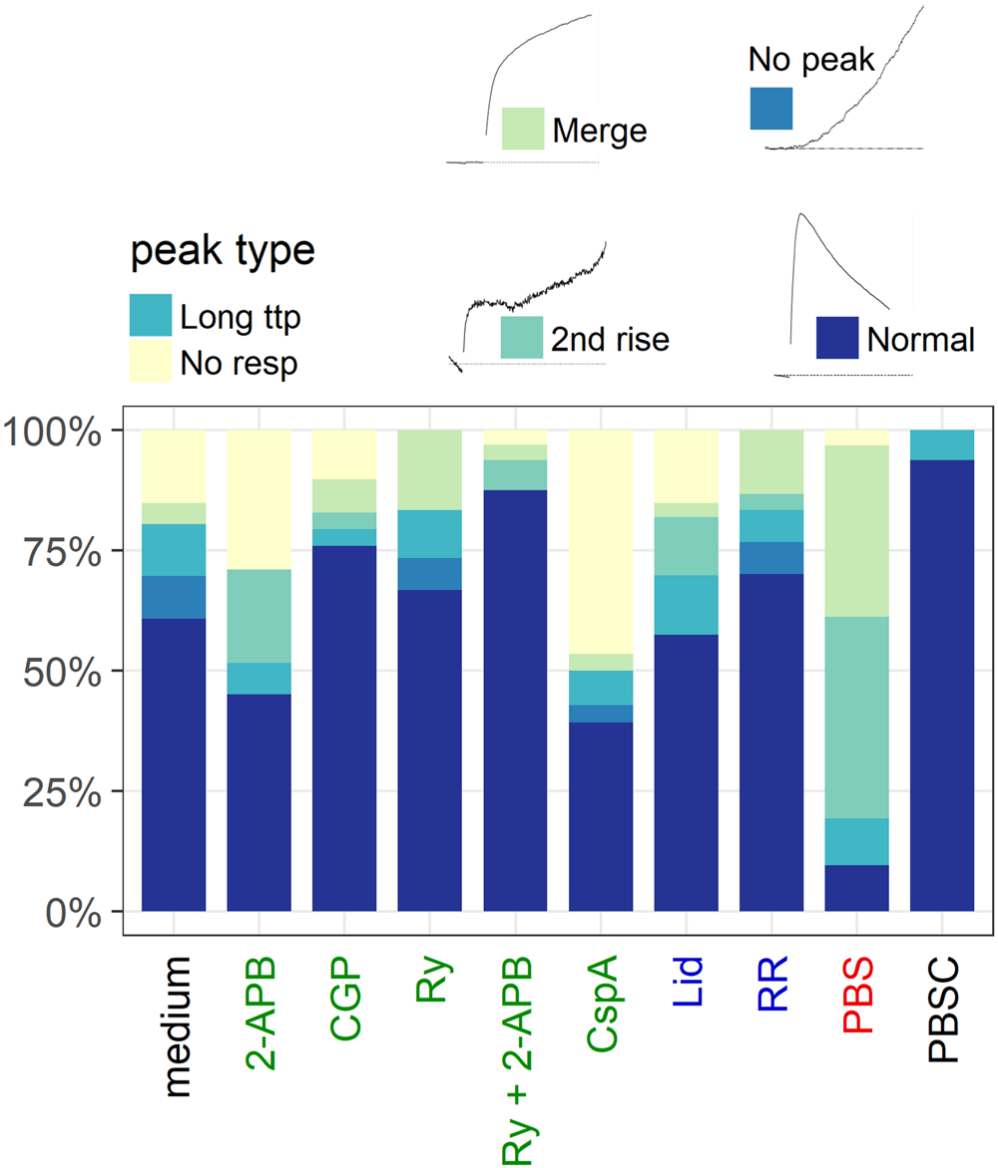
Summary of different calcium signatures in N2A cells by condition. Label colors refer to the targeted mechanism of calcium transport (see Fig. 2). Representative depictions of the different calcium signatures are shown for reference. Long ttp describes cells for which the time to peak did not fall in the respective experiment**’**s interquartile range and that were therefore categorized as outliers. Under certain conditions, the normal calcium signature was observed less frequently. In PBS, 77.5**%** of cells showed either a merge or a second rise. 46**%** of cells in CspA exhibited no response.

Absence of extracellular calcium (Ca^2+^-free PBS) had the largest impact on ∆F/F_0_. Figure 5 (a,c) shows the average change in fluorescence for each experiment divided by experimental condition. When PBS was used instead of culture medium, N2A cells displayed a markedly reduced calcium signal. The same was observed for MCN in PBS compared to PBSC. The inhibitors CGP, ryanodine and especially CspA appeared to decrease the overall calcium response in N2A cells. PI influx was evaluated to assess membrane damage, as membrane perforation constitutes a way for Ca^2+^ to enter the cell independently of its inherent pathways. When only PI-negative cells are considered, cells treated with 2-APB or ryanodine displayed a weaker calcium response than cells that were treated only with culture medium (Fig. 5b). In CspA, the number of responding PI-negative cells per experiment was too low (<3) to calculate an average value for ∆F/F_0_. A comparison of peak fluorescence values of all PI-positive and negative cells is depicted in Fig. 5d. In the presence of 2-APB, ryanodine or both, cells exhibited a markedly stronger response if PI-influx occurred as well. Without inhibitors, PI-positive cells exhibit a larger variation, but the overall increase of peak fluorescence values is less pronounced. Time to peak does not show considerable variation (data not shown).Apart from the quantitative measurements, the different experimental conditions had an impact on the shape of the calcium signature in N2A cells, implying different mechanisms, kinetics, or both. Representative depictions of the different shapes and a summary of their occurrence by condition are given in Fig. 6. At a radiant exposure of 25 mJ/cm^2^, 61% of N2A cells in culture medium displayed a normal calcium response as shown in Fig. 3a, while 15% were not activated by the laser stimulus. Overall, cells in PBS deviated the most from the normal calcium signature, which was observed in only 10% of targeted cells. 35.5% experienced a second increase after a first peak, and 42% showed a merge. The remaining 9.5% represent outliers in time to peak. The highest proportion of non-responding cells is found among those treated with CspA (46%). Averaged across the whole data set, N2A cells showing a normal calcium response experienced a mean fluorescence increase of ∆F/F_0_ = 301 ± 187%. If the first peak was followed by a second rise, the mean peak value was 23 ± 25.5%. Where the immediate rise directly merged into a slower increase, the estimated average peak value was 58 ± 66.3%. PI-positive and negative cells appeared to be distributed evenly across the different types of calcium traces.

Notably, MCN only showed a second fluorescence increase when kept in PBS (33.3%). All other cells displayed a normal calcium trace. A slow onset followed by a linear rise (no peak) was never observed.

### Lipid peroxidation

Lipid peroxidation in the cell membrane was investigated with a fluorescent lipid peroxidation sensor (BODIPY). Oxidation events in the membrane constitute stress signals for the cell and are often a result of excessive ROS production in the cell^26^.

**Figure 7:**
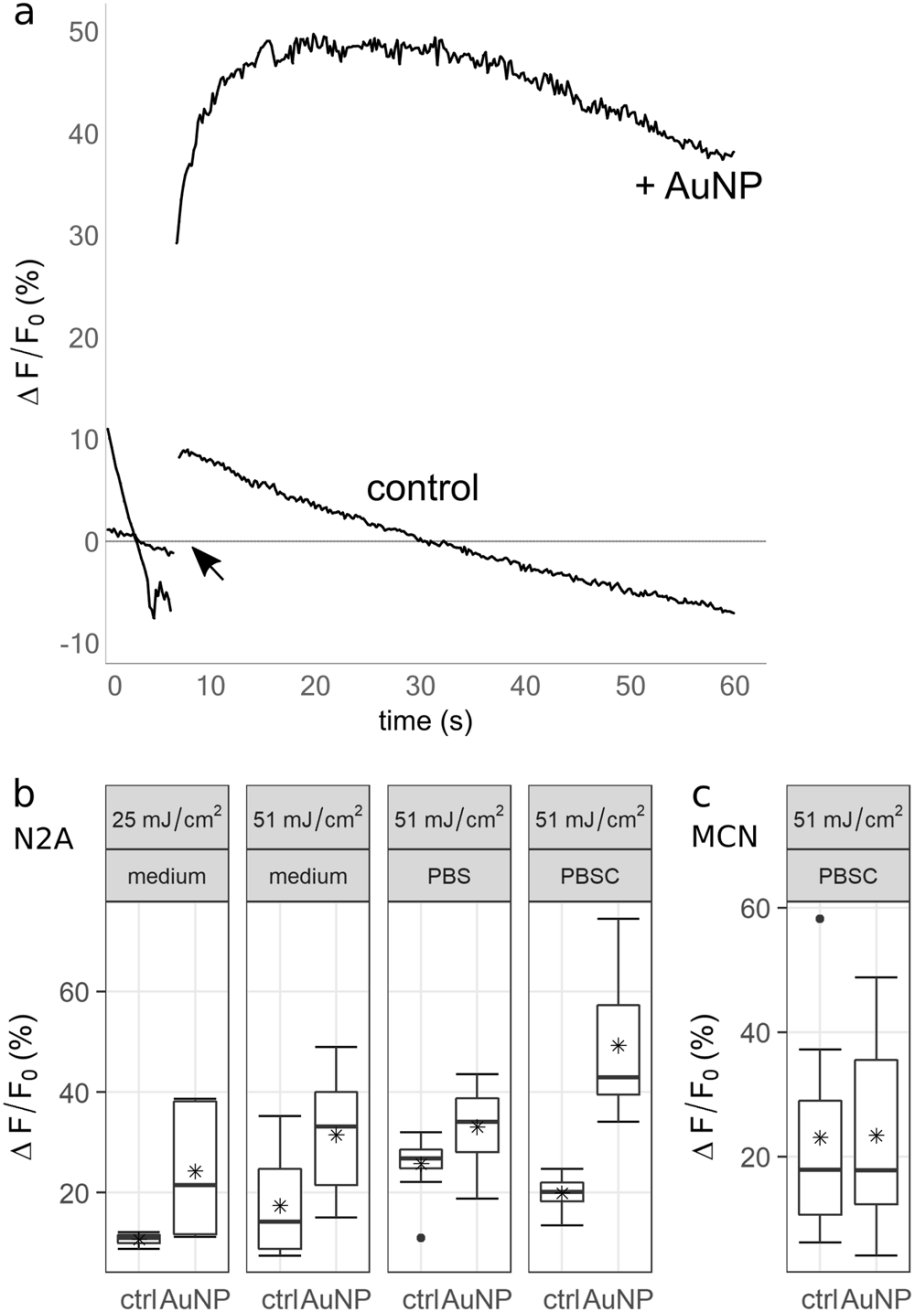
Fluorescence signals and peak fluorescence values for the green-emitting (oxidized) form of BODIPY 581/591 after laser stimulation. Control experiments were performed without gold nanoparticles. (**a**) Representative traces of fluorescence increase over time in N2A cells after laser stimulation with and without AuNP. In the presence of nanoparticles, the dye**’**s fluorescence increase was both faster and more pronounced. (**b**,**c**) In N2A cells, ∆F/F_0_ values increased in the presence of AuNP. No distinction can be made by power. PBSC might however strengthen the oxidizing effect. Lipidperoxidation in MCN was only assessed at 51 mJ/cm². In these cells, the oxidation by the laser alone appears to be of a similar magnitude as in the presence of AuNP.

In the absence of AuNP, the laser stimulus alone evoked a fast increase of BODIPY fluorescence at 520 nm in the majority of cells (96.4%). However, cells that were pre-treated with AuNP showed a markedly stronger and slower increase under all experimental conditions (culture medium, PBS, PBSC), indicating a stronger oxidation process. Figure 7 illustrates the obtained fluorescence signals and the resulting ∆F/F_0_ values of the green emitting form of BODIPY 581/591. The decrease in fluorescence is likely to be the result of photobleaching. No differences became apparent that could be attributed to the different media or the radiant exposure. Notably, the effect was considerably smaller in MCN. Moreover, fluorescence spontaneously increased in eight N2A cells in PBS, i.e. the onset of the increase was not immediately connected to the laser pulse. These spontaneous events occurred both in the presence and absence of AuNP, after a laser-induced fluorescence increase.

## Discussion

Our study shows that a short laser pulse in the presence of gold nanoparticles evokes a substantial calcium response in a neuronal cell line as well as in primary cortical neurons. These cellular reactions have the following key characteristics: 1) Their calcium signature is highly reproducible; 2) peak cytosolic calcium concentrations are reached within five to ten seconds; 3) radiant exposure above a certain threshold does not influence peak values, but is negatively correlated with time to peak; 4) in the absence of extracellular calcium, the calcium response has a lower amplitude and is often followed by a secondary increase of cytosolic calcium.

Ca^2+^ is an important messenger in the cellular stress response and can be both a consequence and a source of cell stress^13,27^. The immediate reaction and the magnitude of the cells’ response to a laser stimulus clearly indicate that laser treatment inflicts considerable stress upon the cells. Since the reaction was absent in AuNP-free control cells, the effect can be attributed to the interactions between the excited nanoparticles and the cell membrane. As a consequence of these interactions, membrane perforation can occur depending on radiant exposure, as was demonstrated by influx of PI or other small molecules in this and previous studies^18–20^ (Fig. 4e). The strength of the stressor, i.e. the increase in radiant exposure, was reflected in the decreasing time to peak.

The overall time course of the calcium signal and the lack of an effect of the inhibitor lidocaine exclude action potentials as the main underlying cause. The calcium trace expected from electrophysiological activity reaches its maximum considerably faster, on a millisecond timescale^28^. The smooth and reproducible rise of cytosolic calcium contradicts the expectations from a burst of action potentials, were fast calcium transients add up to a rather irregular pattern^29^.

It has been shown before that upon membrane perforation, a volume exchange between a cell and the surrounding medium takes place^15,30^. This exchange was described to be initially fast and to slow down with time. It has therefore been hypothesized that calcium flux through transient membrane pores might be a main source of strong calcium responses like those observed in the present study^15,30^. Cells in calcium-free PBS still showed calcium signals in response to laser stimulation, proving the involvement of at least one intracellular source. Those signals were however noticeably weaker and often altered in shape in comparison to cells in culture medium (Figs 5, 6). To assess the role of calcium influx following membrane perforation, a thorough analysis of the whole data set (738 N2A cells, 169 MCN) was performed.

In PI-negative cells, the inhibitors 2-APB and ryanodine, both of which target calcium channels in the ER-membrane, appeared to decrease the amplitude of the calcium response (Fig. 5b). Furthermore, both inhibitors caused a clear distinction between peak calcium values of PI-positive and negative cells (Fig. 5d). The propidium ion that is responsible for the nucleotide staining of perforated cells is considerably larger than the hydrated Ca^2+^ ion (1.5 nm^31^ compared to approx. 412 pm^32^). Therefore, Ca^2+^ might enter the cell through membrane pores even if no PI signal is detectable. It has been argued however, that small membrane pores constitute a large energy barrier for positively charged ions which would hinder rapid flux through such defects^33^. Since the immediate calcium response saturated within a few seconds and the calcium responses of PI-positive and negative N2A cells generally exhibited the same features, it seems unlikely that the immediate calcium response is caused primarily by Ca^2+^ influx through membrane defects.

It has been demonstrated before that plasmon excitation of gold nanoparticles in contact with a cellular membrane causes perforation^18–20^. The resulting holes are large enough for small molecules like proteins, RNA or dyes to enter, as is the case in the present study. The respective cells showed good viability in the previous studies, where illumination time was shorter. The overall stable phenotype for minutes after laser stimulation of cells in the present study suggests that even after a 40 ms laser pulse, membrane perforation is only transient. A collapse of stimulated cells was not observed.

The decrease of peak values by the inhibitors of intracellular Ca^2+^ pathways, CspA, 2-APB and ryanodine, support the hypothesis that intracellular calcium release is the key initial response. During a typical calcium response (Fig. 3a), fluorescence increased by about 250% more, on average, than in cases where the initial rise was followed by a second increase. The latter case was particularly often observed in PBS (Fig. 6). This two-component calcium response can therefore be considered inherent to the cell: An acute stress signal, represented by laser stimulation of the gold nanoparticles in contact with the cell membrane, evokes Ca^2+^ outflow from the ER. This outflow is initially fast, and might transition to a slower, more sustained Ca^2+^ release. In some cases, the fast phase was omitted and the stimulus evoked only a slow, linear calcium outflow. Such slow kinetics of Ca^2+^ release from the ER have been described before as a response to oxidative stress^34^. If the initial response is strong enough however, the slow phase is no longer observed – possibly because it is overshadowed by the strong calcium signal. The large increase in the cytosolic Ca^2+^ concentration is likely to stem from various sources. Of the tested inhibitors, only CspA strongly decreases the calcium signal. Together with the aforementioned lack of variability of calcium signatures in culture medium, this leads us to hypothesize the involvement of the mitochondrial membrane permeability transition pore (mMPTP), which is blocked by CspA^35,36^. This megachannel spans both mitochondrial membranes and opens in response to high levels of mitochondrial stress, caused, amongst other stressors, by calcium overload. When the initial rise of cytosolic calcium is strong enough, large amounts of calcium are sequestered by the mitochondria, which in turn undergo membrane permeability transition. This is reflected by the observations that 2-APB and ryanodine can decrease the calcium signal when no substantial influx from the surrounding medium occurs, i.e. in PI-negative cells (Fig. 5b). Only CspA consistently decreases the signal almost to levels of cells in Ca^2+^-free PBS (Fig. 5a). Influx from the extracellular space might still take place, but calcium flow through the mMPTP is hindered. Next to the opening of the megachannel, mitochondrial calcium overload leads to a disruption of the membrane potential and subsequent ATP consumption^14^, ROS production^37^ and mitochondrial swelling and possibly ruptures^37,38^.

Integrating all information gained from our observations (magnitude, time course, shape and responsiveness to modulators of the calcium response) and extensive comparisons with the available literature allow us to present conclusive evidence that gold nanoparticle mediated laser stimulation of a neuronal cell line constitutes a potent stressor for a cell, inducing Ca^2+^ release from the ER, opening of the mMPTP and thereby a sustained increase of cytosolic calcium concentrations. Extracellular calcium entering through membrane defects plays a role in potentiating the initial stress signal and calcium overload of the mitochondria, but is not the primary source of rising cytosolic calcium.

Notably, MCN never experience an increase of intracellular calcium at a comparable extent as N2A cells. While various factors complicate a direct comparison, it is possible that different cell types possess different levels of susceptibility to the stressor that is the laser stimulus. While the mechanisms underlying the hypothesized stress response are universal, the extent of their activation might vary across cell types and their environments. This is also reflected by the lack of MCN activation in their culture medium.

Stimulating cells stained with BODIPY 581/591 showed that in the presence of AuNP, the laser stimulus caused membrane lipids to oxidize (Fig. 7). The process of oxidation usually includes formation of lipid radicals and reactive oxygen species (ROS)^39,40^. These reactive molecules are known to damage numerous cellular macromolecules if present at high levels. They constitute an unspecific stress signal and might represent a way for the cell to sense lipid oxidation or molecule breakdown occurring in the vicinity of irradiated AuNP. Dejeans *et al*.^34^ demonstrated that oxidative stress causes a slow, sustained calcium release from the ER, agreeing with the continually increasing Ca^2+^ levels sometimes observed after an initial fast response or instead of a fast response (see Fig. 6, “2nd rise” and “No peak”). This further reinforces our hypothesis: if the initial fast response is not present or is particularly weak, mitochondrial Ca^2+^ levels don’t rise high enough to cause opening of the mMPTP and subsequent high cytosolic calcium levels. Instead, a milder form of ER stress emerges, as is triggered by oxidative stress^34^. This is consistent with the observation that in MCN, a weaker calcium response occurs in combination with a less pronounced lipid peroxidation process. Whether or not a cell experiences the profound and potentially fatal calcium stress response as described above might therefore depend on the membrane’s susceptibility for lipid damage caused by the external stimulus. This susceptibility is likely to be altered by the cell’s immediate environment – i.e. adjacent cells, tissue biology or medium composition.

## Conclusion

In this study, we highlighted for the first time the consequences that gold nanoparticle mediated laser stimulation can have for neuronal cells. The lack of studies concerned with the metabolic impact of laser stimulation made it necessary for us to first collect a large amount of unbiased data reflecting (patho-) physiological processes triggered by the stimulus. Visualizing calcium flux and lipid peroxidation in a large number of cells allowed us to formulate a coherent hypothesis: Irradiation of AuNP at their plasmon resonance frequency triggers calcium release from the ER, often accompanied and potentiated by calcium inflow through a perforated membrane. The excess calcium is sequestered by the mitochondria, and in response to calcium overload, permeability transition sets in. Subsequent studies will be necessary to specifically determine the involvement of the mMPTP. Regardless of the source however, calcium overload of a cell generally has adverse effects, especially if repeated stimulation leads to chronic cell stress. Extensive impacts on cell physiology and metabolism are the consequences, linked for example to different neurodegenerative diseases^41,42^. In *in vivo* applications, a healthy biological interface is crucial for functionality of a neuroimplant. Our results highlight the problematic nature of a safe translation of this application into living organisms, although the specific cell response might vary in complex tissues. Since the general approach allows for cell stimulation with high spatiotemporal precision it could prove to be a valuable tool in cell biology, enabling for example the study of long-term effects of stressors affecting only single cells in a network, cellular priming by triggering a calcium signal, or the complex interplay of physiological and pathophysiological pathways.
